# Disparities and Gaps in Breast Cancer Screening for Women Aged 40 to 49 Years

**DOI:** 10.1001/jamanetworkopen.2024.51827

**Published:** 2024-12-20

**Authors:** Tianshu Gu, Jing Yuan, Shelley White-Means, Minghui Li

**Affiliations:** 1 College of Graduate Health Sciences, University of Tennessee Health Science Center, Memphis; 2 Department of Clinical Pharmacy, Fudan University, Shanghai, China; 3 Department of Interprofessional Education, University of Tennessee Health Science Center, Memphis; 4 Department of Clinical Pharmacy and Translational Science, University of Tennessee Health Science Center, Memphis

## Abstract

This cross-sectional study analyzes factors associated with overdue or lack of mammogram screening among women aged 40 to 49 years.

## Introduction

The US Preventive Services Task Force (USPSTF) recently released recommendations for breast cancer screening, advising biennial mammography screening for all women aged 40 to 74 years.^1^ This marks a substantial shift from the previous recommendation for women in their 40s, transitioning from an individual decision to begin screening.^2^ This adjustment aims to advance early breast cancer detection and address inequity in breast cancer mortality, particularly among Black women.^3^ This study aimed to examine disparities and gaps in breast cancer screening among women aged 40 to 49 years.

## Methods

This cross-sectional study was approved by the University of Tennessee Health Science Center institutional review board with a waiver of informed consent (due to the use of deidentified data) and followed the STROBE reporting guideline. We utilized nationally representative data from the National Health Interview Survey in 2019 and 2021.^4^ The study population comprised women aged 40 to 49 years who had not previously been diagnosed with breast cancer. Populations examined included racial and ethnic minority populations, sexual minority populations, rural residents, individuals with disabilities, and socioeconomically disadvantaged populations.

Mammography screening was self-reported and classified into 3 groups: those who had undergone a mammogram within the past 2 years (biennial screening), those who had undergone a mammogram more than 2 years ago (overdue screening), and those who had never received a mammogram (no screening). Multinomial logistic regression analysis was performed to identify factors associated with overdue and no mammography screening. To ensure accurate representation and address the complex survey design, survey sampling weights were applied in all analyses. The threshold for statistical significance was a 2-sided *P* < .05. Analyses were conducted using SAS software version 9.4 (SAS Institute) from June to October 2024. See the eMethods in Supplement 1 for more information.

## Results

Among 20.1 million women aged 40 to 49 years, 11.7 million (weighted percentage, 59.2%) reported having undergone mammography screening within the last 2 years, 3.0 million (weighted percentage, 15.2%) reported having had a screening more than 2 years ago, and 5.0 million (weighted percentage, 25.6%) reported having never undergone mammography screening. Biennial screening rates were significantly lower among several minority groups: non-Hispanic women with other race (ie, American Indian or Alaska Native alone, American Indian or Alaska Native and another racial or ethnic group, and any other racial and ethnic group not specified), lesbian and bisexual women, rural residents, and those with a family income at 138% or less of the federal poverty level (FPL) (Figure). The rate of biennial screening decreased as family income decreased.

**Figure.**
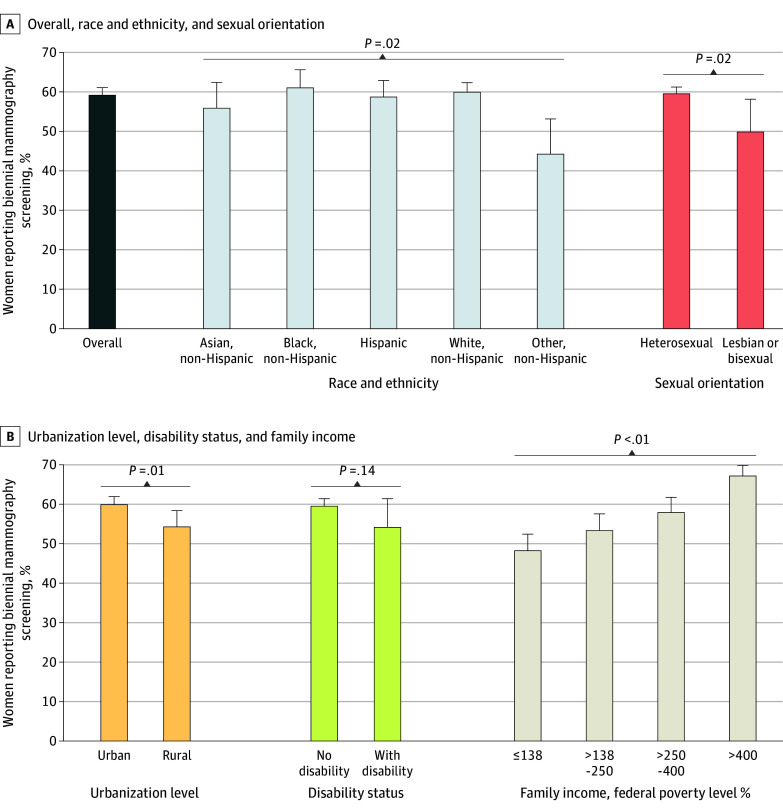
Percentage of Women Aged 40 to 49 Years Reporting Biennial Mammography Screening by Race and Ethnicity, Sexual Orientation, Urbanization Level, Disability Status, and Family Income The other, non-Hispanic group includes women who identified as non-Hispanic American Indian or Alaska Native only, non-Hispanic American Indian or Alaska Native and any other racial or ethnic group, and any single and/or multiple race and ethnicity group not otherwise specified.

Compared with biennial mammography screening, lacking a usual place of care was significantly associated with a higher likelihood of overdue screening (risk difference [RD], 0.07; 95% CI, 0.01-0.14) (Table). Factors associated with a higher probability of no screening included being non-Hispanic Asian (RD, 0.09; 95% CI, 0.01-0.18), family income based on FPL (≤138%: RD, 0.07; 95% CI, 0.01-0.14; >138%-250%: RD, 0.10; 95% CI, 0.04-0.16; >250%-400%: RD, 0.09; 95% CI, 0.04-0.15), being uninsured (RD, 0.13; 95% CI, 0.06-0.21), and lacking a usual place for care (RD, 0.20; 95% CI, 0.13-0.28). The absence of a usual place for care was associated with both overdue and no mammography screening.

**Table.** Factors Associated With Overdue or No Mammography Screening Among Women Aged 40 to 49 Years

Characteristic	Risk difference (95% CI)	
	Overdue screening vs biennial screening^a^	No screening vs biennial screening^a^
Race and ethnicity		
Asian, non-Hispanic	−0.04 (−0.11 to 0.03)	0.09 (0.01 to 0.18)
Black, non-Hispanic	−0.03 (−0.09 to 0.03)	−0.04 (−0.10 to 0.03)
Hispanic	−0.04 (−0.10 to 0.01)	−0.06 (−0.12 to 0.01)
White, non-Hispanic	0 [Reference]	0 [Reference]
Other, non-Hispanic^b^	0.02 (−0.10 to 0.14)	0.04 (−0.07 to 0.15)
Sexual orientation		
Heterosexual	0 [Reference]	0 [Reference]
Lesbian or bisexual	0.07 (−0.03 to 0.16)	0.02 (−0.07 to 0.12)
Urbanization level		
Urban	0 [Reference]	0 [Reference]
Rural	0.03 (−0.03 to 0.09)	−0.01 (−0.08 to 0.05)
Disability status		
No	0 [Reference]	0 [Reference]
Yes	0.01 (−0.07 to 0.09)	−0.03 (−0.13 to 0.06)
Family income, federal poverty level %		
≤138	0.04 (−0.03 to 0.11)	0.07 (0.01 to 0.14)
>138-250	−0.01 (−0.06 to 0.04)	0.10 (0.04 to 0.16)
>250-400	−0.02 (−0.07 to 0.03)	0.09 (0.04 to 0.15)
>400	0 [Reference]	0 [Reference]
Health status		
Fair or poor	0.04 (−0.02 to 0.10)	−0.03 (−0.10 to 0.03)
Good, very good, excellent	0 [Reference]	0 [Reference]
Marital status		
Married	0 [Reference]	0 [Reference]
Divorced or widowed	0.04 (−0.01 to 0.09)	−0.01 (−0.06 to 0.04)
Never married	0.00 (−0.05 to 0.06)	0.05 (−0.01 to 0.11)
Education level		
Less than high school	0 [Reference]	0 [Reference]
High school	0.00 (−0.07 to 0.08)	0.02 (−0.07 to 0.11)
Some college	0.04 (−0.04 to 0.11)	−0.01 (−0.10 to 0.07)
College and above	0.01 (−0.07 to 0.08)	−0.05 (−0.14 to 0.05)
Region		
Northeast	0 [Reference]	0 [Reference]
Midwest	0.04 (−0.03 to 0.10)	−0.02 (−0.10 to 0.05)
South	0.03 (−0.02 to 0.09)	−0.01 (−0.08 to 0.06)
West	0.01 (−0.05 to 0.08)	0.07 (0.00 to 0.14)
Employment		
No	−0.01 (−0.05 to 0.04)	0.04 (−0.01 to 0.10)
Yes	0 [Reference]	0 [Reference]
Health insurance		
Private	0 [Reference]	0 [Reference]
Medicaid or other public	−0.04 (−0.10 to 0.03)	0.05 (−0.02 to 0.12)
Other coverage	−0.04 (−0.14 to 0.06)	−0.07 (−0.18 to 0.04)
Uninsured	0.05 (−0.02 to 0.12)	0.13 (0.06 to 0.21)
Usual place for care		
No	0.07 (0.01 to 0.14)	0.20 (0.13 to 0.28)
Yes	0 [Reference]	0 [Reference]

^a^ Reference group in the multinomial logistic regression.

^b^ The Other, non-Hispanic group includes non-Hispanic American Indian or Alaska Native only, non-Hispanic American Indian or Alaska Native and any other racial or ethnic group, and any single and/or multiple race and ethnicity group not otherwise specified.

## Discussion

The findings of this cross-sectional study underscore significant disparities and gaps in biennial mammography screening for women aged 40 to 49 years. Two-fifths of women in this age group did not receive biennial screening. Lower biennial screening rates were observed among racial and ethnic minority populations, sexual minority populations, rural residents, and socioeconomically disadvantaged populations. To optimize early breast cancer detection, ensuring equitable adherence to USPSTF recommendations is crucial.^5^ This study found similar mammography screening rates between Black and White women. To reduce racial disparities in breast cancer mortality, efforts should focus on addressing delays and ensuring guideline-concordant treatment.^6^

This study identified both unique and shared factors associated with overdue or no mammography screening. To effectively implement USPSTF recommendations, particular emphasis should be placed on addressing the needs of women without a usual place for care. This study is limited in that data were collected during a period when biennial mammography screening was considered an individual decision. Targeted interventions and policies aimed at enhancing health care access, coverage, and affordability hold significant potential to improve health equity in biennial mammography screening for women aged 40 to 49 years.

## Notes

Supplement 1.
eMethods

Supplement 2.
Data Sharing Statement
