# Supplementary material for: Disparities and Gaps in Breast Cancer Screening for Women Aged 40 to 49 Years
Source: JAMA Netw Open. 2024 Dec 20;7(12):e2451827. doi: 10.1001/jamanetworkopen.2024.51827 (PMC11662252; doi:10.1001/jamanetworkopen.2024.51827)
Supplement: Supplement 1. — eMethods [file jamanetwopen-e2451827-s001.pdf]

Supplemental Online Content

Gu T, Yuan J, White-Means S, Li M. Disparities and gaps in breast cancer screening for women aged 40 to 49 years. *JAMA Netw. Open*. 2024;7(12):e2451827. doi:10.1001/jamanetworkopen.2024.51827

eMethods.

This supplemental material has been provided by the authors to give readers additional information about their work.

© 2024 Gu T et al. *JAMA Network Open.*

eMethods

Race and ethnicity is self-reported in the National Health Interview Survey (NHIS). Race and ethnicity were queried in the same survey question: "Single and multiple race groups with Hispanic origin." All race and ethnicity options include: Hispanic, non-Hispanic White only, non-Hispanic Black or African American only, non-Hispanic Asian only, and non-Hispanic other (defined as non-Hispanic American Indian or Alaska Native only, non-Hispanic American Indian or Alaska Native and any other group, and any single and/or multiple race and ethnicity group not otherwise specified). We assessed race and ethnicity in the study because we are examining racial and ethnic minorities and included race and ethnicity as factors in the multinomial logistic regression.

© 2024 Gu T et al. *JAMA Network Open.*
