# Supplementary material for: Disparities and Gaps in Breast Cancer Screening for Women Aged 40 to 49 Years
Source: JAMA Netw Open. 2024 Dec 20;7(12):e2451827. doi: 10.1001/jamanetworkopen.2024.51827 (PMC11662252; doi:10.1001/jamanetworkopen.2024.51827)
Supplement: Supplement 2. — Data Sharing Statement [file jamanetwopen-e2451827-s002.pdf]

Data Sharing Statement

Gu. Disparities and Gaps in Breast Cancer Screening for Women Aged 40 to 49 Years. *JAMA Netw Open*. Published December 20, 2024. doi:10.1001/jamanetworkopen.2024.51827

Data
Data available: No

Additional Information
Explanation for why data not available: We utilized nationally representative data from the National Health Interview Survey (NHIS). These data are publicly available from the Centers for Disease Control and Prevention (CDC) website at: https://www.cdc.gov/nchs/nhis/
